# Supplementary material for: Unraveling the deep learning gearbox in optical coherence tomography image segmentation towards explainable artificial intelligence
Source: Commun Biol. 2021 Feb 5;4:170. doi: 10.1038/s42003-021-01697-y (PMC7864998; doi:10.1038/s42003-021-01697-y)
Supplement: Supplementary file 2 — Description of Additional Supplementary Files [file 42003_2021_1697_MOESM2_ESM.pdf]

Description of Additional Supplementary Files

Files: Supplementary Data 1-5

Description: All source data underlying the graphs and charts presented in the main figures.
