# Supplementary material for: Unraveling the deep learning gearbox in optical coherence tomography image segmentation towards explainable artificial intelligence
Source: Commun Biol. 2021 Feb 5;4:170. doi: 10.1038/s42003-021-01697-y (PMC7864998; doi:10.1038/s42003-021-01697-y)
Supplement: Supplementary file 8 — Reporting Summary [file 42003_2021_1697_MOESM8_ESM.pdf]

nature research

Corresponding author(s): Peter M Maloca NMETH-A42868

Last updated by author(s): Jun 15, 2020

Reporting Summary

Nature Research wishes to improve the reproducibility of the work that we publish. This form provides structure for consistency and transparency in reporting. For further information on Nature Research policies, see our Editorial Policies and the Editorial Policy Checklist.

Statistics

For all statistical analyses, confirm that the following items are present in the figure legend, table legend, main text, or Methods section.

n/a	Confirmed	
☐	☒	The exact sample size (n) for each experimental group/condition, given as a discrete number and unit of measurement
☐	☒	A statement on whether measurements were taken from distinct samples or whether the same sample was measured repeatedly
☐	☒	The statistical test(s) used AND whether they are one- or two-sided<br>*Only common tests should be described solely by name; describe more complex techniques in the Methods section.*
☒	☐	A description of all covariates tested
☐	☒	A description of any assumptions or corrections, such as tests of normality and adjustment for multiple comparisons
☐	☒	A full description of the statistical parameters including central tendency (e.g. means) or other basic estimates (e.g. regression coefficient) AND variation (e.g. standard deviation) or associated estimates of uncertainty (e.g. confidence intervals)
☐	☒	For null hypothesis testing, the test statistic (e.g. F, t, r) with confidence intervals, effect sizes, degrees of freedom and P value noted<br>*Give P values as exact values whenever suitable.*
☒	☐	For Bayesian analysis, information on the choice of priors and Markov chain Monte Carlo settings
☒	☐	For hierarchical and complex designs, identification of the appropriate level for tests and full reporting of outcomes
☒	☐	Estimates of effect sizes (e.g. Cohen's d, Pearson's r), indicating how they were calculated

Our web collection on statistics for biologists contains articles on many of the points above.

Software and code

Policy information about availability of computer code

Data collection	Data collection was performed in animals manually using the manufacturer's device and software.
Data analysis	* Python, v3.7.4; numpy, v1.16.4, pillow, v6.1.0: used for ground truth augmentation and ground truth preparation for CNN training<br>* Python, v3.7.4; tensorflow, v1.14: used for CNN training and predicting with fully-trained CNNs<br>* Python, v3.7.4; pandas, v0.25.1; numpy, v1.16.5; matplotlib, v3.1.1: used for Hamming distance calculation, multidimensional scaling and plotting figures 2 and 3<br>* C#, v7.3; .NET Framework, v4.6: used for calculating 3D volumes of compartments Vitreous, Retina, Choroid, and Sclera<br>* R (programming language), v3.6.1; R-package perm, v1.0-0.0: used for statistical analyses

For manuscripts utilizing custom algorithms or software that are central to the research but not yet described in published literature, software must be made available to editors and reviewers. We strongly encourage code deposition in a community repository (e.g. GitHub). See the Nature Research guidelines for submitting code & software for further information.

Data

Policy information about availability of data

All manuscripts must include a data availability statement. This statement should provide the following information, where applicable:

- Accession codes, unique identifiers, or web links for publicly available datasets
- A list of figures that have associated raw data
- A description of any restrictions on data availability

Some authors (SN, TKS, TS, ND) are bound by confidentiality agreements and data restrictions, so that the OCT imaging data cannot be shared.

Field-specific reporting

Please select the one below that is the best fit for your research. If you are not sure, read the appropriate sections before making your selection.

- [x] Life sciences
- [ ] Behavioural & social sciences
- [ ] Ecological, evolutionary & environmental sciences

For a reference copy of the document with all sections, see nature.com/documents/nr-reporting-summary-flat.pdf

Life sciences study design

All studies must disclose on these points even when the disclosure is negative.

Sample size	Sample size was calculated with regard to another report: 19. Faulkner L. Beyond the five-user assumption: benefits of increased sample sizes in usability testing. Behav Res Methods Instrum Comput. 2003; 35: 379–383.
Data exclusions	No data were excluded.
Replication	The reproducibility of the method was published previously in thie paper: Validation of automated artificial intelligence segmentation of optical coherence tomography images. Maloca PM, Lee AY, de Carvalho ER, Okada M, Fasler K, Leung I, Hörmann B, Kaiser P, Suter S, Hasler PW, Zarranz-Ventura J, Egan C, Heeren TFC, Balaskas K, Tufail A, Scholl HPN. PLoS One. 2019 Aug 16;14(8):e0220063. doi: 10.1371/journal.pone.0220063. eCollection 2019.
Randomization	The ten eyes for annotation stage 'tutorial set' were selected by random sampling without replacement. For each of the ten eyes in 'tutorial set', ten OCT B-scans were chosen by random sampling without replacement. A total of forty eyes for 'test set' and 'CNN training and validation set' were chosen by random sampling without replacement and subsequently split into 'test set' (8 eyes) and 'CNN training and validation set' (32 eyes) by random sampling. For random sampling, sort (GNU coreutils), v8.28 was used.
Blinding	All graders performed their grading independently and blinded as they used a personalized and password protected remote account.

Reporting for specific materials, systems and methods

We require information from authors about some types of materials, experimental systems and methods used in many studies. Here, indicate whether each material, system or method listed is relevant to your study. If you are not sure if a list item applies to your research, read the appropriate section before selecting a response.

Materials & experimental systems

n/a	Involved in the study	
x		Antibodies
x		Eukaryotic cell lines
x		Palaeontology and archaeology
	x	Animals and other organisms
x		Human research participants
x		Clinical data
x		Dual use research of concern

Methods

n/a	Involved in the study	
x		ChIP-seq
x		Flow cytometry
x		MRI-based neuroimaging

Animals and other organisms

Policy information about studies involving animals; ARRIVE guidelines recommended for reporting animal research

Laboratory animals	cynomolgus monkeys of Mauritian genetic background
Wild animals	n/a
Field-collected samples	n/a
Ethics oversight	Data for this study were acquired during the baseline examinations of routine pharmaceutical product development studies and thus no additional animals were used specifically for the purpose of this study. Animal care and experimentation were conducted in accordance with the Association for Assessment and Accreditation of Laboratory Animal Care international (AAALACi) and the Canadian Council on Animal Care (CCAC) guidelines or the guidelines of the US National Research Council, respectively. The original protocols have been reviewed and approved by the Institutional Animal Care and Use Committee (CRL Montreal IACUC, respectively Covance Madison IACUC)

Note that full information on the approval of the study protocol must also be provided in the manuscript.
